# Supplementary material for: Mortality risks from a spectrum of causes associated with sand and dust storms in China
Source: Nat Commun. 2023 Oct 27;14:6867. doi: 10.1038/s41467-023-42530-w (PMC10611721; doi:10.1038/s41467-023-42530-w)
Supplement: Supplementary file 1 — Supplementary Information [file 41467_2023_42530_MOESM1_ESM.pdf]

Supplementary Information for

Mortality risks from a spectrum of causes associated with sand and dust storms in China

Total Pages: 16
Number of Tables: 1
Number of Figures: 10

Supplementary Methods

To date, there is no uniform standard for defining Sand and Dust Storms (SDS) events. Several studies used the local official sand-dust weather records in the study area for SDS events definition[1–12]. Official sand-dust weather records usually have the advantages of authority and accuracy, but most countries do not have unified sand-dust weather records, and researchers often need to compile records from various data sources themselves[11]. Moreover, the records often do not meet the research needs for the spatial and temporal resolution of sand-dust weather record information; for example, China's official sand-dust weather records are only recorded at the provincial level.

Thus, some researchers have set a series of indicators based on the physicochemical characteristics of sand-dust weather, such as anomalies in particulate matter concentration and composition, anomalies in meteorological factors and weather records, and developed a series of definition methods for SDS events by combining multiple indicators (Table S1)[13–37]. Among these indicators, PM_{10} concentration and $PM_{2.5}/PM_{10}$ concentration ratio are relatively important judgment indicators in existing studies because the data are readily available and can reflect the critical physical characteristics of sand-dust weather.

In this study, considering data accessibility and accuracy of results, a combination of multiple indicators, including PM_{10} concentrations and $PM_{2.5}/PM_{10}$ concentration ratio, based on official sand-dust weather records was used to define SDS events.

Table S1 Indicators for sand and dust storms events definition used in existing literature.

Indicator Dimension	Indicator	Examples	Strengths	Limitations
Anomalies in particulate matter concentration	PM_{10}[13,15–19,21,23–25,28–30,35,36]	Hourly concentration >150μg/m³ or daily average concentration >50 μg/m³	It is the main physical characteristic of sand–dust weather, and relevant data are available.	There is no uniform standard threshold.
	$PM_{2.5}$/ PM_{10}[18,38–40]	$PM_{2.5}$/ PM_{10} concentration ratio < 0.4	It is the main physical characteristic of sand–dust weather, and relevant data are available.	There is no uniform standard threshold.
	AQI (Air Quality Index)[38]	AQI > 100	Relevant data are available.	Not a typical feature of sand-dust weather.
Anomalies in particulate matter composition	Crustal elements[41]	Elements such as Al are at least 3 times higher than the usual average value	It is the main physical characteristic of sand–dust weather.	Poor data accessibility.
	Anthropogenic components[41]	As, Zn, Cu, Pb, sulfate, nitrate, organic carbon, low values of elemental carbon	It is the main physical characteristic of sand–dust weather.	Poor data accessibility.
Anomalies in meteorological factors	Visibility[13,14,17,19,20,33]	Ground level visibility <10 km	It is the main physical characteristic of sand–dust weather.	Poor data accessibility.
	Wind speed[13,26]	Ground wind speed >17m/s	It is the main physical characteristic of serious sand–dust weather.	Fail to recognize sand–dust weather of low intensity, such as floating dust, sand blowing weather.
Anomalies in weather records	Weather observation records of nearby airports[9,13]	Record of aircraft grounding at nearby airports due to sand–dust weather	High accuracy.	Poor data accessibility.
	News Media Record[11]	News reporting on the occurrence of sand–dust weather	Relevant data are easily available.	Variable quality, often used for verification of identification results.

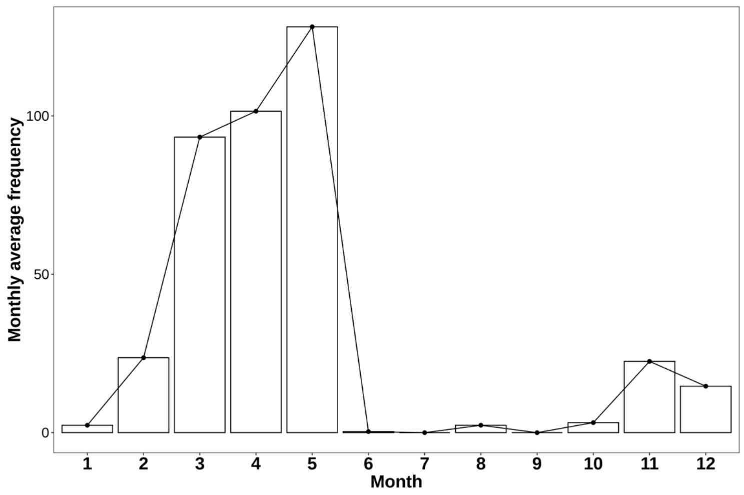

Fig. S1. The monthly average frequency of sand and dust storms events in the study counties between 2013 and 2018.

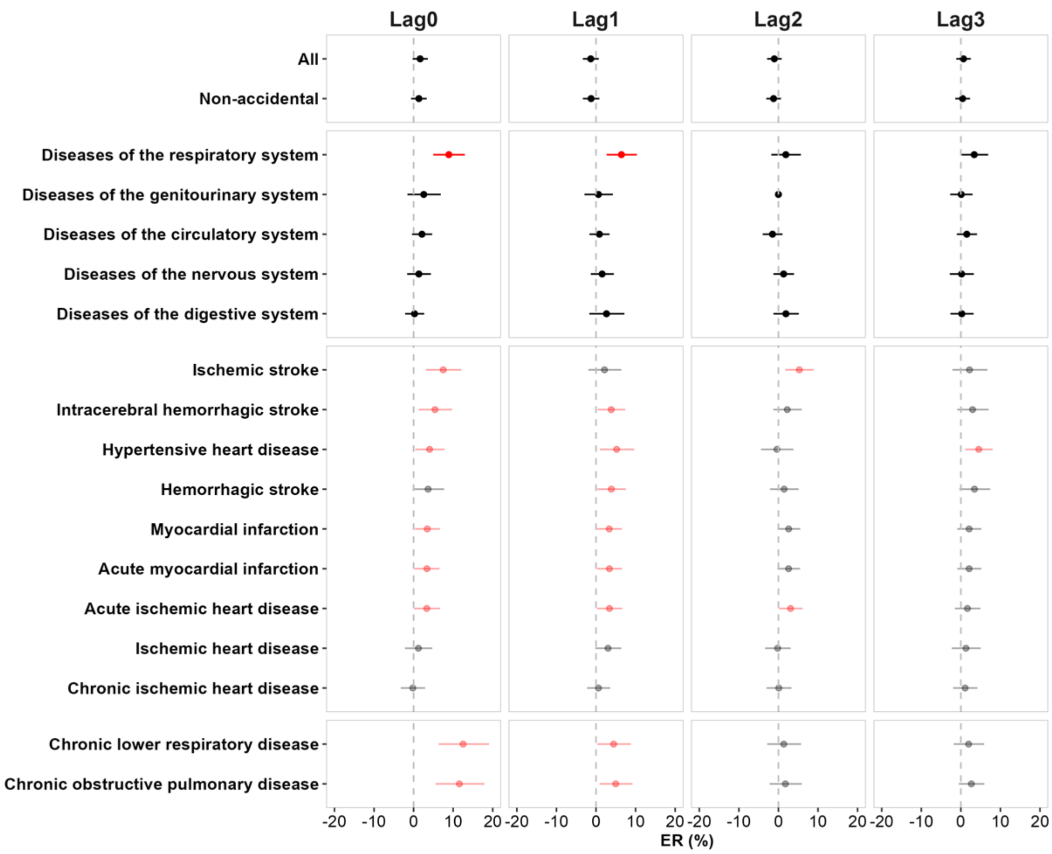

Fig. S2. Lag-specific mortality risk associated with sand and dust storms events. Points represent the estimated excess risk (ER, %). Horizontal lines represent the 95% confidence interval (CI). Red represents that the ER differed significantly from 0% (P < 0.05). The Mortality of broad causes results are shown in the top two panels, and mortality of specific causes results in the bottom two panels.

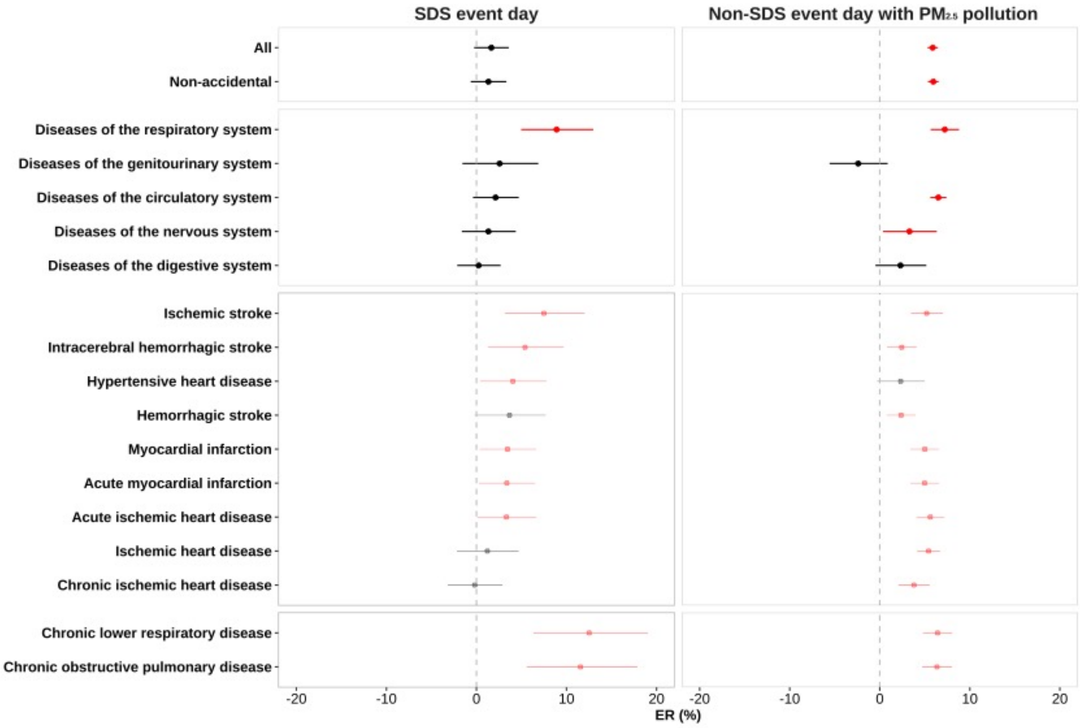

Fig. S3. Mortality risk associated with Sand and Dust Storms (SDS) event day (left) or non-SDS event day with $PM_{2.5}$ pollution (right). $PM_{2.5}$ pollution represents that daily $PM_{2.5}$ concentration ≥75 μg/m^3. Points represent the estimated excess risk (ER, %). Horizontal lines represent the 95% confidence interval (CI). Red represents that the ER differed significantly from 0% ($P < 0.05$). The Mortality of broad causes results are shown in the top two panels, and mortality of specific causes results in the bottom two panels.

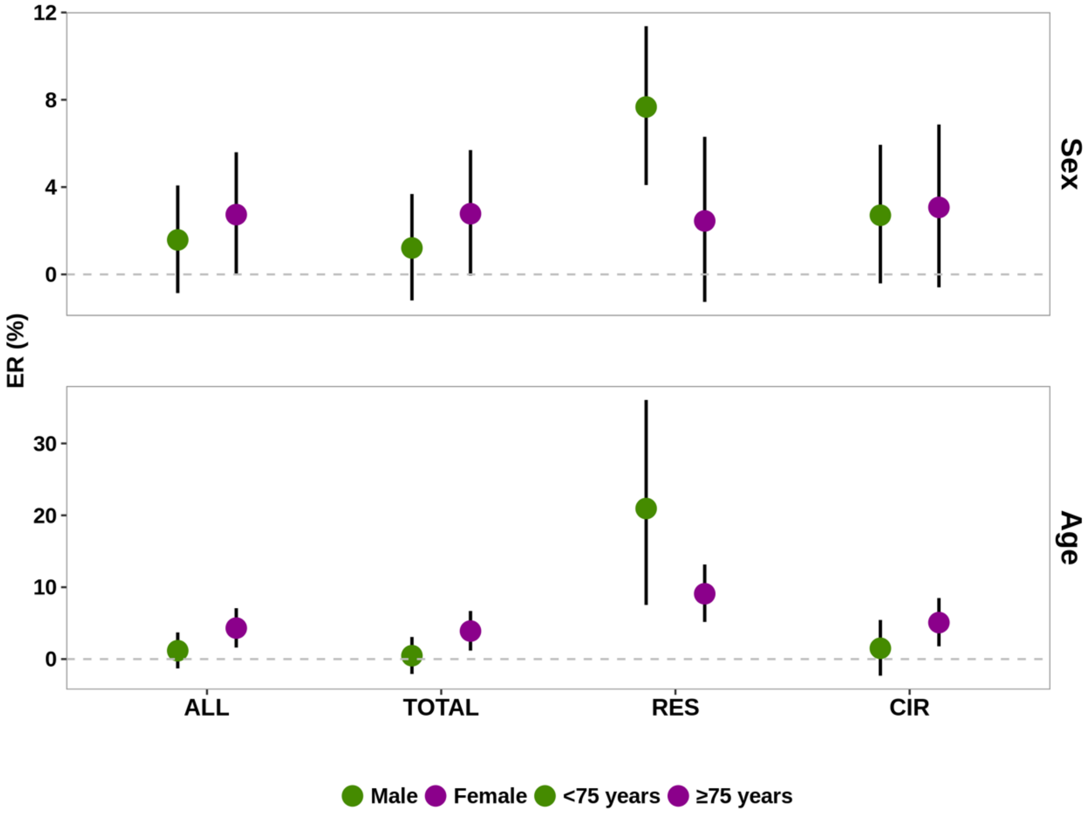

Fig. S4. Mortality risk associated with sand and dust storms events in sex groups (top panel) and age groups (bottom panel). ALL: all-cause mortality; TOTAL: non-accidental mortality; RES: mortality due to respiratory system diseases; CIR: mortality due to circulatory system diseases. Points represent the estimated excess risk (ER, %). Vertical lines represent the 95% confidence interval (CI). No significant differences were observed in the estimates by sex- and age groups based on the Z test.

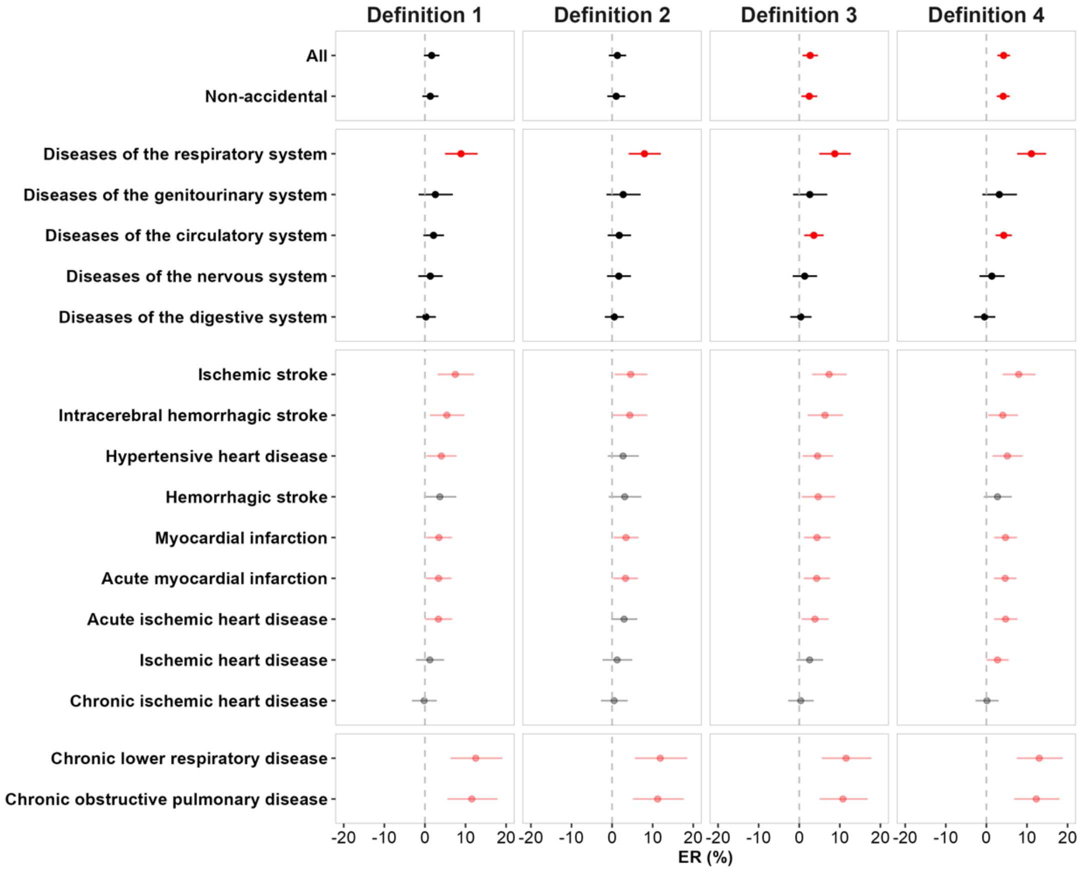

Fig. S5. Mortality risk associated with Sand and Dust Storms (SDS) events under different definitions of SDS events. "Definition 1" represents estimates under the primary SDS events definition with the $PM_{2.5}/PM_{10}$ concentration ratio <0.4; "Definition 2" and "Definition 3" represent estimates under the alternative definitions of SDS events with the $PM_{2.5}/PM_{10}$ concentration ratio less than 0.35 and 0.45, respectively. "Definition 4" represents estimates under an alternative definition of SDS events without considering the $PM_{2.5}/PM_{10}$ concentration ratio. All the estimates were from our primary analytical models. Points represent the estimated excess risk (ER, %). Horizontal lines represent the 95% confidence interval (CI). Red represents that the ER differed significantly from 0% ($P < 0.05$). The Mortality of broad causes results are shown in the top two panels, and mortality of specific causes results in the bottom two panels.

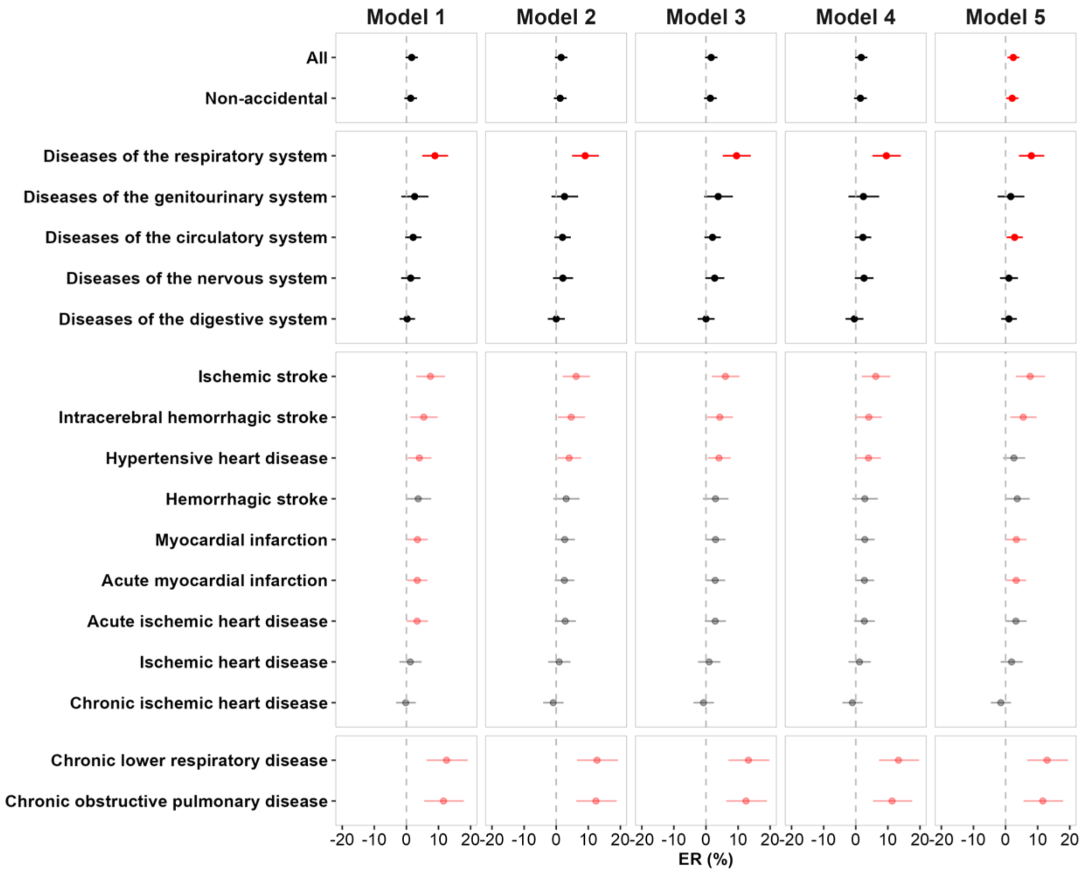

Fig. S6. Mortality risk associated with sand and dust storms events using different model settings. "Model 1" represents estimates from the primary model, with the degree of freedom (df) of 2 and 3, for time variable and meteorological parameters in natural spline functions. "Model 2" represents estimates from the primary model, except the df for the time variable was 3. "Model 3" represents estimates from the primary model, except the df for the time variable was 3, and the dfs for meteorological parameters were 4. "Model 4" represents estimates from the primary model, except the df for the time variable was 3, and the dfs for meteorological parameters were 5. "Model 5" represents estimates from the primary model, except using a 21-day moving average of temperature and a 7-day moving average of relative humidity. Points represent the estimated excess risk (ER, %). Horizontal lines represent the 95% confidence interval (CI). Red represents that the ER differed significantly from 0% ($P < 0.05$). The Mortality of broad causes results are shown in the top two panels, and mortality of specific causes results in the bottom two panels.

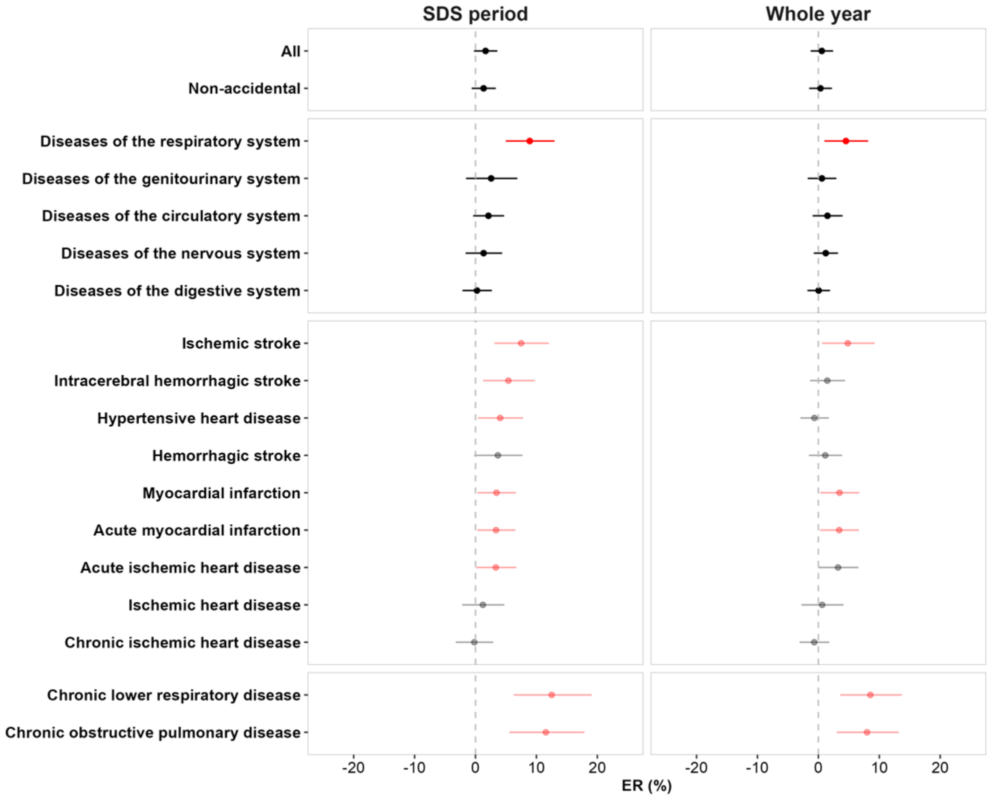

Fig. S7. Mortality risk associated with Sand and Dust Storms (SDS) events based on models fit with different study period. "SDS period" represents estimates from our primary analysis conducted during the SDS period (1 February–31 May), 2013-2018. "Whole year" represents estimates from the main model equation conducted during the whole year, 2013-2018. Points represent the estimated excess risk (ER, %). Horizontal lines represent the 95% confidence interval (CI). Red represents that the ER differed significantly from 0% ($P < 0.05$). The Mortality of broad causes results are shown in the top two panels, and mortality of specific causes results in the bottom two panels.

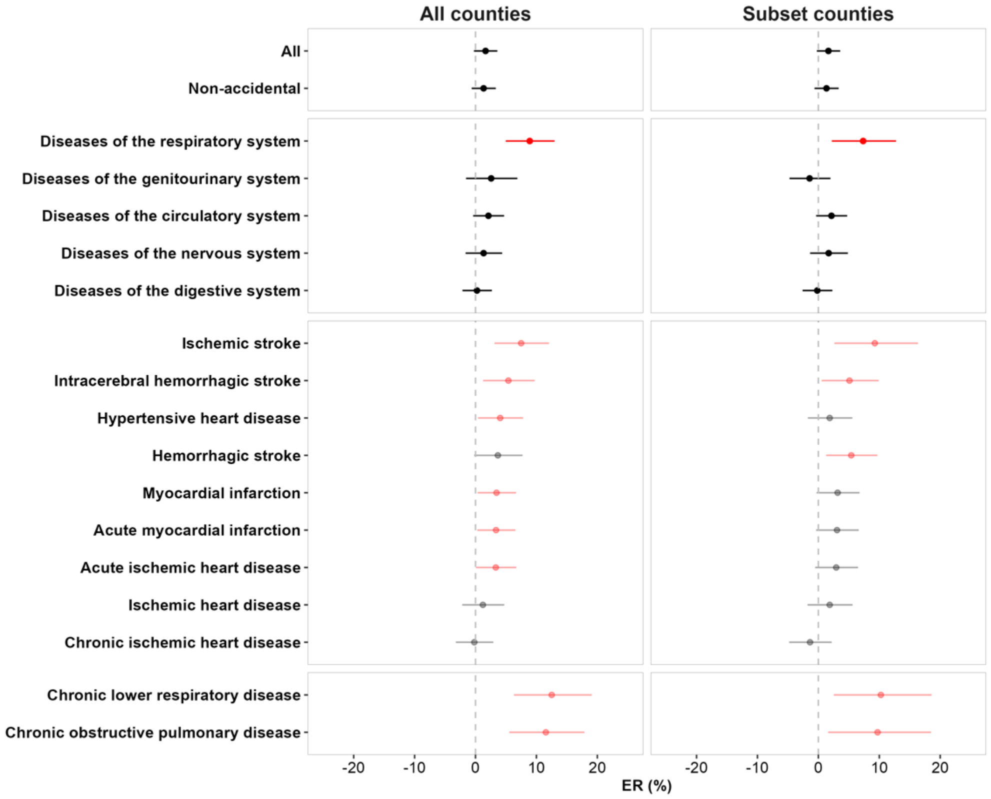

Fig. S8. Mortality risk associated with Sand and Dust Storms (SDS) events based on models fit using data of different study counties. “All counties” represents estimates from our primary analysis using data for all study counties. “Subset counties” represents estimates from the sensitivity analysis excluding counties with an average daily death count of less than one during the SDS period (1 February–31 May), 2013-2018. The number of counties used in the sensitivity analysis ranged from 44 to 194. Points represent the estimated excess risk (ER, %). Horizontal lines represent the 95% confidence interval (CI). Red represents that the ER differed significantly from 0% ($P < 0.05$). The Mortality of broad causes results are shown in the top two panels, and mortality of specific causes results in the bottom two panels.

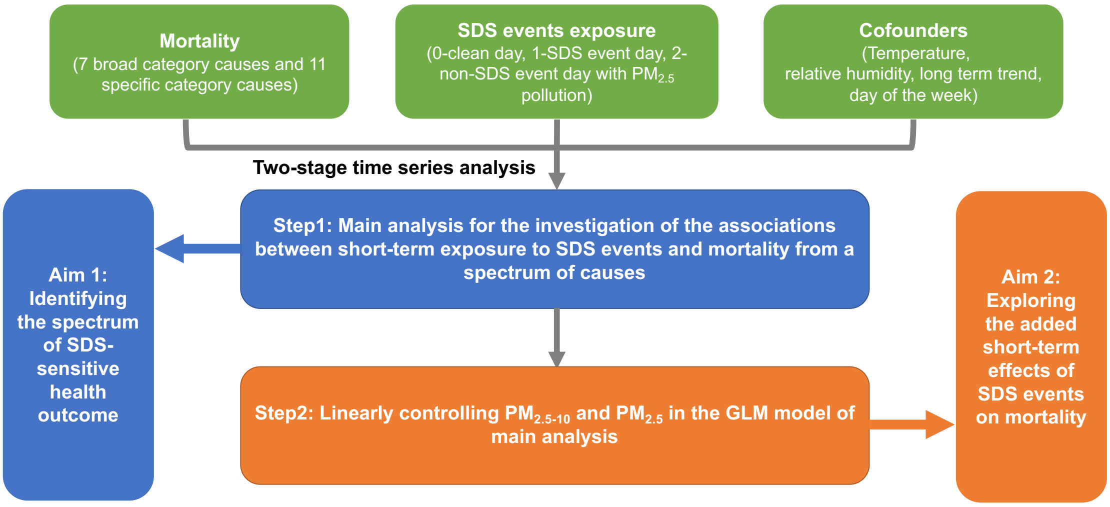

Fig. S9. Study design and the procedures of data analyses. SDS: sand and dust storms.

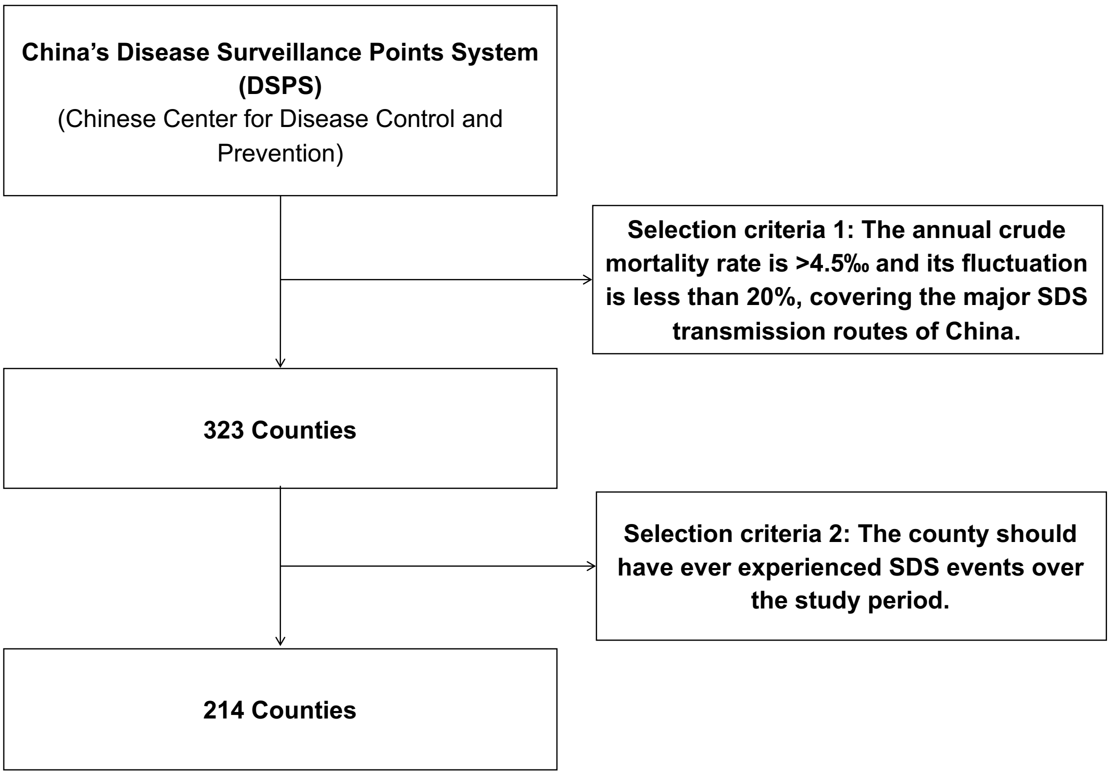

Fig. S10. The selection procedure for the study counties. SDS: sand and dust storms.

Supplementary References

1. Chan, C.-C. & Ng, H.-C. A case-crossover analysis of Asian dust storms and mortality in the downwind areas using 14-year data in Taipei. *Sci. Total Environ.* **410–411**, 47–52 (2011).

2. Kojima, S. *et al.* Asian dust exposure triggers acute myocardial infarction. *Eur. Heart J.* **38**, 3202–3208 (2017).

3. Jung, J. *et al.* Burden of dust storms on years of life lost in Seoul, South Korea: a distributed lag analysis. *Environ. Pollut.* **296**, 118710 (2022).

4. Matsukawa, R. *et al.* Desert dust is a risk factor for the incidence of acute myocardial infarction in western Japan. *Circ. Cardiovasc. Qual. Outcomes* **7**, 743–748 (2014).

5. Meng, Z. & Lu, B. Dust events as a risk factor for daily hospitalization for respiratory and cardiovascular diseases in Minqin, China. *Atmos. Environ.* **41**, 7048–7058 (2007).

6. Lee, H., Kim, H., Honda, Y., Lim, Y.-H. & Yi, S. Effect of Asian dust storms on daily mortality in seven metropolitan cities of Korea. *Atmos. Environ.* **79**, 510–517 (2013).

7. Kwon, H.-J., Cho, S.-H., Chun, Y., Lagarde, F. & Pershagen, G. Effects of the Asian dust events on daily mortality in Seoul, Korea. *Environ. Res.* **90**, 1–5 (2002).

8. Wang, Y.-C. & Lin, Y.-K. Mortality associated with particulate concentration and Asian dust storms in Metropolitan Taipei. *Atmos. Environ.* **117**, 32–40 (2015).

9. Neophytou, A. M. *et al.* Particulate matter concentrations during desert dust outbreaks and daily mortality in Nicosia, Cyprus. *J. Expo. Sci. Environ. Epidemiol.* **23**, 275–280 (2013).

10. Kamouchi, M. *et al.* Relationship between Asian dust and ischemic stroke: a time-stratified case-crossover study. *Stroke* **43**, 3085–3087 (2012).

11. Crooks, J. L. *et al.* The association between dust storms and daily non-accidental mortality in the United States, 1993–2005. *Environ. Health Persp.* **124**, 1735–1743 (2016).

12. Kim, H.-S., Kim, D.-S., Kim, H. & Yi, S.-M. Relationship between mortality and fine particles during Asian dust, smog–Asian dust, and smog days in Korea. *Int. J. Environ. Health Res.* **22**, 518–530 (2012).

13. Middleton, N. *et al.* A 10-year time-series analysis of respiratory and cardiovascular morbidity in Nicosia, Cyprus: the effect of short-term changes in air pollution and dust storms. *Environ. Health* **7**, 39 (2008).

14. Ishii, M. *et al.* Association of short term exposure to Asian dust with increased blood pressure. *Sci. Rep.* **10**, 17630 (2020).

15. Stafoggia, M. *et al.* Desert dust outbreaks in southern Europe: contribution to daily PM_{10} concentrations and short-term associations with mortality and hospital admissions. *Environ. Health Persp.* **124**, 413–419 (2016).

16. Samoli, E., Kougea, E., Kassomenos, P., Analitis, A. & Katsouyanni, K. Does the presence of desert dust modify the effect of PM_{10} on mortality in Athens, Greece? *Sci. Total Environ.* **409**, 2049–2054 (2011).

17. Lee, H. *et al.* Effect of Asian dust storms on mortality in three Asian cities. *Atmos. Environ.* **89**, 309–317 (2014).

18. Tam, W. W. S., Wong, T. W. & Wong, A. H. S. Effect of dust storm events on daily emergency admissions for cardiovascular diseases. *Circ. J.* **76**, 655–660 (2012).

19. Aghababaeian, H. *et al.* Effect of dust storms on non-Accidental, cardiovascular, and respiratory mortality: a case of dezful city in Iran. *Environ. Health Insights* **15**, 11786302211060152 (2021).

20. Lee, S. *et al.* Effects of Asian dust-derived particulate matter on ST-elevation myocardial infarction: retrospective, time series study. *BMC Public Health* **21**, 68 (2021).

21. Johnston, F., Hanigan, I., Henderson, S., Morgan, G. & Bowman, D. Extreme air pollution events from bushfires and dust storms and their association with mortality in Sydney, Australia 1994–2007. *Environ. Res.* **111**, 811–816 (2011).

22. Zhang, Q., Zhang, J., Yang, Z., Zhang, Y. & Meng, Z. Impact of $PM_{2.5}$ derived from dust events on daily outpatient numbers for respiratory and cardiovascular diseases in Wuwei, China. *Procedia Environ. Sci.* **18**, 290–298 (2013).

23. Domínguez-Rodríguez, A. *et al.* Impact of Saharan dust on the incidence of acute coronary syndrome. *Rev. Esp. Cardiol.* **74**, 321–328 (2021).

24. Vodonos, A. *et al.* Individual effect modifiers of dust exposure effect on cardiovascular morbidity. *PLoS One* **10**, e0137714 (2015).

25. Teng, J. C.-Y., Chan, Y.-S., Peng, Y.-I. & Liu, T.-C. Influence of Asian dust storms on daily acute myocardial infarction hospital admissions. *Public Health Nurs.* **33**, 118–128 (2016).

26. Ho, H. C., Wong, M. S., Yang, L., Chan, T.-C. & Bilal, M. Influences of socioeconomic vulnerability and intra-urban air pollution exposure on short-term mortality during extreme dust events. *Environ. Pollut.* **235**, 155–162 (2018).

27. Jiménez, E., Linares, C., Martínez, D. & Díaz, J. Role of Saharan dust in the relationship between particulate matter and short-term daily mortality among the elderly in Madrid (Spain). *Sci. Total Environ.* **408**, 5729–5736 (2010).

28. Zauli Sajani, S. *et al.* Saharan dust and daily mortality in Emilia-Romagna (Italy). *Occup. Environ. Med.* **68**, 446–451 (2011).

29. Perez, L. *et al.* Saharan dust, particulate matter and cause-specific mortality: a case–crossover study in Barcelona (Spain). *Environ. Int.* **48**, 150–155 (2012).

30. Al-Taiar, A. & Thalib, L. Short-term effect of dust storms on the risk of mortality due to respiratory, cardiovascular and all-causes in Kuwait. *Int. J. Biometeorol.* **58**, 69–77 (2014).

31. Ma, Y. *et al.* Short-term effects of air pollution on daily hospital admissions for cardiovascular diseases in western China. *Environ. Sci. Pollut. Res.* **24**, 14071–14079 (2017).

32. Renzi, M. *et al.* Short-term effects of desert and non-desert PM_{10} on mortality in Sicily, Italy. *Environ. Int.* **120**, 472–479 (2018).

33. JROAD Investigators *et al.* Short-term exposure to desert dust and the risk of acute myocardial infarction in Japan: a time-stratified case-crossover study. *Eur. J. Epidemiol.* **35**, 455–464 (2020).

34. Hwang, S. S. *et al.* The Asian dust events and hospital admissions with respiratory and cardiovascular disease in Seoul, Korea: Isee-249. *Epidemiology* **14**, S48 (2003).

35. Byun, G., Kim, H., Choi, Y. & Lee, J.-T. The difference in effect of ambient particles on mortality between days with and without yellow dust events: using a larger dataset in Seoul, Korea from 1998 to 2015. *Sci. Total Environ.* **691**, 819–826 (2019).

36. Bell, M. L., Levy, J. K. & Lin, Z. The effect of sandstorms and air pollution on cause-specific hospital admissions in Taipei, Taiwan. *Occup. Environ. Med.* **65**, 104–111 (2008).

37. Tchounwou, P. The effects of $PM_{2.5}$ from Asian dust storms on emergency room visits for cardiovascular and respiratory diseases. *Int. J. Environ. Res. Public Health* **14**, 428 (2004).

38. Tam, W. W. S., Wong, T. W., Wong, A. H. S. & Hui, D. S. C. Effect of dust storm events on daily emergency admissions for respiratory diseases. *Respirology* **17**, 143–148 (2012).

39. Zhao, D., Chen, H., Yu, E. & Luo, T. $PM_{2.5}$ /PM_{10} ratios in eight economic regions and their relationship with meteorology in China. *Adv. Meteorol.* **2019**, 1–15 (2019).

40. Filonchyk, M. Characteristics of the severe March 2021 Gobi Desert dust storm and its impact on air pollution in China. *Chemosphere* **287**, 132219 (2022).

41. Tong, D. Q., Wang, J. X. L., Gill, T. E., Lei, H. & Wang, B. Intensified dust storm activity and Valley fever infection in the southwestern United States. *Geophys. Res. Lett.* **44**, 4304–4312 (2017).
