# Supplementary material for: Mortality risks from a spectrum of causes associated with sand and dust storms in China
Source: Nat Commun. 2023 Oct 27;14:6867. doi: 10.1038/s41467-023-42530-w (PMC10611721; doi:10.1038/s41467-023-42530-w)
Supplement: Supplementary file 3 — Reporting Summary [file 41467_2023_42530_MOESM3_ESM.pdf]

nature portfolio

Corresponding author(s): Tiantian Li

Last updated by author(s): Aug 11, 2023

Reporting Summary

Nature Portfolio wishes to improve the reproducibility of the work that we publish. This form provides structure for consistency and transparency in reporting. For further information on Nature Portfolio policies, see our Editorial Policies and the Editorial Policy Checklist.

Statistics

For all statistical analyses, confirm that the following items are present in the figure legend, table legend, main text, or Methods section.

n/a	Confirmed	
☐	☒	The exact sample size (*n*) for each experimental group/condition, given as a discrete number and unit of measurement
☒	☐	A statement on whether measurements were taken from distinct samples or whether the same sample was measured repeatedly
☐	☒	The statistical test(s) used AND whether they are one- or two-sided<br>*Only common tests should be described solely by name; describe more complex techniques in the Methods section.*
☐	☒	A description of all covariates tested
☐	☒	A description of any assumptions or corrections, such as tests of normality and adjustment for multiple comparisons
☐	☒	A full description of the statistical parameters including central tendency (e.g. means) or other basic estimates (e.g. regression coefficient) AND variation (e.g. standard deviation) or associated estimates of uncertainty (e.g. confidence intervals)
☐	☒	For null hypothesis testing, the test statistic (e.g. *F*, *t*, *r*) with confidence intervals, effect sizes, degrees of freedom and *P* value noted<br>*Give P values as exact values whenever suitable.*
☒	☐	For Bayesian analysis, information on the choice of priors and Markov chain Monte Carlo settings
☒	☐	For hierarchical and complex designs, identification of the appropriate level for tests and full reporting of outcomes
☒	☐	Estimates of effect sizes (e.g. Cohen's *d*, Pearson's *r*), indicating how they were calculated

Our web collection on statistics for biologists contains articles on many of the points above.

Software and code

Policy information about availability of computer code

Data collection	No software was used for data collection.
Data analysis	We provide a sample of the code used in this project online at https://github.com/sunshineann/SDS_mortality_NatureComm.

For manuscripts utilizing custom algorithms or software that are central to the research but not yet described in published literature, software must be made available to editors and reviewers. We strongly encourage code deposition in a community repository (e.g. GitHub). See the Nature Portfolio guidelines for submitting code & software for further information.

Data

Policy information about availability of data

All manuscripts must include a data availability statement. This statement should provide the following information, where applicable:

- Accession codes, unique identifiers, or web links for publicly available datasets
- A description of any restrictions on data availability
- For clinical datasets or third party data, please ensure that the statement adheres to our policy

The data generated in this study are available under restricted access for the identifiable nature of the data and data management requirements. Access can be obtained by contacting the corresponding author (litiantian@nieh.chinacdc.cn) and will be answered within 12 weeks. The data can be used through collaborative research with authors. The exposure data for air pollution in this study was available from China's National Air Pollution Monitoring System (http://www.cnemc.cn) upon request. The exposure data for meteorological data in this study were downloaded from the ERA5-land reanalysis dataset released by European Centre for

Medium-Range Weather Forecasts (https://cds.climate.copernicus.eu/cdsapp#!/dataset/reanalysis-era5-land?tab=overview). Official sand–dust weather records were collected from the Sand-dust Weather Almanac compiled by the China Meteorological Administration, a book published by the Meteorological Publishing House. The electronic version of this book can be downloaded from the China Knowledge Network (https://www.cnki.net). Source data are provided with this paper.

Research involving human participants, their data, or biological material

Policy information about studies with human participants or human data. See also policy information about sex, gender (identity/presentation), and sexual orientation and race, ethnicity and racism.

Reporting on sex and gender	In addition to our primary analysis, we conducted a stratified analysis based on sex. We used sex rather than gender because sex is the available variable as collected in the mortality dataset. We provide full results for these stratified models in Figure S4.
Reporting on race, ethnicity, or other socially relevant groupings	n/a
Population characteristics	n/a
Recruitment	n/a
Ethics oversight	Our study obtained approvals (Chinese Environmental Public Health Tracking and Risk Assessment, 202102) from the ethics committee of the National Institute of Environmental Health, Chinese Center for Disease Control and Prevention (NIEH, China CDC).

Note that full information on the approval of the study protocol must also be provided in the manuscript.

Field-specific reporting

Please select the one below that is the best fit for your research. If you are not sure, read the appropriate sections before making your selection.

☐ Life sciences ☐ Behavioural & social sciences ☒ Ecological, evolutionary & environmental sciences

For a reference copy of the document with all sections, see nature.com/documents/nr-reporting-summary-flat.pdf

Ecological, evolutionary & environmental sciences study design

All studies must disclose on these points even when the disclosure is negative.

Study description	This is a multicenter, nationwide study in China, to extensively investigate the associations between Sand and Dust Storms (SDS) events exposure and daily mortality from a spectrum of causes. We first performed a two-stage time series analysis using the daily data from 2013 to 2018 for 214 Chinese counties. Further, we investigated the added effects of SDS events by controlling PM2.5-10 and PM2.5 in the models, respectively.
Research sample	From 2013 to 2018, a total of 1,495,724 deaths records during the SDS period were collected from the China's Disease Surveillance Points System of the Chinese Center for Disease Control and Prevention. This national mortality dataset represents the general population in China well.
Sampling strategy	No sample size calculation was performed. We used a nationwide dataset which is sufficient to represent the general population in China.
Data collection	Data used in this study were collected and organized by CC, TL, and CZ. Daily mortality data were obtained from China's Disease Surveillance Points System of the Chinese Center for Disease Control and Prevention. Daily county-specific concentrations of air pollutants, including PM10 and PM2.5, were obtained from hourly data reported by China's National Air Pollution Monitoring System. We calculated PM2.5–10 concentration by subtracting PM2.5 concentration from PM10 concentration for each county. Meteorological data, including temperature and relative humidity, were obtained from the ERA5-land reanalysis dataset released by European Centre for Medium-Range Weather Forecasts. Official records of sand–dust weather used to identify sand and dust storms event were collected from Sand-dust Weather Almanac compiled by China Meteorological Administration.
Timing and spatial scale	Due to data accessibility, data were collected from January 1, 2013 to December 31, 2018. Regarding the spatial scale, data from 214 Chinese counties were included, the primary SDS transmission routes, with a good representation of the heterogeneity in exposure levels to SDS events.
Data exclusions	No data were excluded from the analyses.
Reproducibility	We conducted a series of sensitivity analyses to assess the robustness of the estimated associations between SDS events and many mortality outcomes in our primary analysis. First, we examined the mortality risk of SDS events under different SDS definitions. Second, we changed the df for the time trend variable (df = 3) and used two different df (df = 4, 5) for meteorological parameters in the spline functions. Third, instead of the daily mean temperature and relative humidity, we used the 21-day moving average of temperature and the 7-day moving average of relative humidity to fully adjust for the confounding of meteorological conditions61.

Fourth, we refit the GLM model in the first stage using the whole year's data. Fifth, since the daily county-level death counts were pretty small for certain mortality outcomes, we conducted sensitivity analyses only on study counties with daily death counts exceeding one during the SDS periods. This approach allowed us to examine the potential uncertainty introduced by these low counts. All models gave consistent information regarding our primary message about the estimates.

Randomization	n/a. This study is an observational study.
Blinding	n/a. This study is an observational study.

Did the study involve field work? ☐ Yes ☒ No

Reporting for specific materials, systems and methods

We require information from authors about some types of materials, experimental systems and methods used in many studies. Here, indicate whether each material, system or method listed is relevant to your study. If you are not sure if a list item applies to your research, read the appropriate section before selecting a response.

Materials & experimental systems

n/a	Involved in the study	
☒	☐	Antibodies
☒	☐	Eukaryotic cell lines
☒	☐	Palaeontology and archaeology
☒	☐	Animals and other organisms
☒	☐	Clinical data
☒	☐	Dual use research of concern
☒	☐	Plants

Methods

n/a	Involved in the study	
☒	☐	ChIP-seq
☒	☐	Flow cytometry
☒	☐	MRI-based neuroimaging
